# Flexible Localized Surface Plasmon Resonance Sensor with Metal–Insulator–Metal Nanodisks on PDMS Substrate

**DOI:** 10.1038/s41598-018-30180-8

**Published:** 2018-08-07

**Authors:** Chiao-Yun Chang, Hsiang-Ting Lin, Ming-Sheng Lai, Teng-Yi Shieh, Chien-Chung Peng, Min-Hsiung Shih, Yi-Chung Tung

**Affiliations:** 1 Research Center of Applied Sciences (RCAS), Academia Sinica, Taipei, 11529 Taiwan; 2 Department of Photonics, National Chiao Tung University (NTCU), Hsinchu, 30010 Taiwan; 3 Department of Photonics, National Sun Yat-sen University (NSYSU), Kaohsiung, 80424 Taiwan

## Abstract

The small sized, flexible, high-performed and bio-compatible sensing devices are the critical elements to realize the bio-related detection or on-site health monitoring systems. In this work, the flexible localized surface plasmon resonance (LSPR) bio-sensors were demonstrated by integrating the metal–insulator–metal (MIM) nanodisks with bio-compatible polydimethylsiloxane (PDMS) substrate. The different geometries of MIM nanodisk sensors were investigated and optimized to enhance the spatial overlap of the LSPR waves with the environment, which lead to a high sensitivity of 1500 nm/RIU. The omni-directional characteristics of LSPR resonances were beneficial for maintaining the device sensitivity stable under various bending curvatures. Furthermore, the flexible MIM nanodisk LSPR sensor was applied to detect A549 cancer cells in PBS+ solution. The absorption peak of the MIM-disk LSPR sensor obviously redshift to easily distinguish between the phosphate buffered saline (PBS+) solution with A549 cancer cells and without cells. Therefore, the flexible MIM nanodisk LSPR sensor is suitable to develop on-chip microfluidic biosensors for detection of cancer cells on nonplanar surfaces.

## Introduction

The growing number of serious diseases caused by ever-increasing pollution has necessitated the use of biosensors in healthcare monitoring systems. Localized surface plasmon resonance (LSPR) biosensing is an optical spectroscopic method that provides real-time, precise and high-sensitivity detection^1–3^. An optical phenomenon, LSPR is caused when incident light induces a locally coherent oscillation of opposite charges at surfaces or interfaces of metals to create surface plasmon resonance (SPR)^4,5^. When the wavelength of the light incident on a metallic nanostructure is considerably larger than that of a plasmonic structure, local oscillations are produced around the nanostructure. This phenomenon can be used for to alter metallic nanostructures. The resonance wavelength (λ) of the LSPR is dependent on the environmental refraction index and geometric shape and structure. LSPR results in a high-intensity and strongly localized electromagnetic field that can be extremely sensitive to even small changes in the surrounding dielectric medium^6^. Regarding the environmental refractive index change (∆n), the shift wavelength (∆λ) of the LSPR is caculated by ∆λ = m∆n^7,8^. Here, m is the sensitivity of the LSPR sensor. By analyzing this LSPR shift, small-sized entities, such as biological antibodies and low-concentration analytes, can be detected^9,10^. This technique can be used for monitoring viruses and cancer cells^11–13^. Thus, LSPR sensors have considerable potential for providing high sensitivity and label-free methods for emerging areas of biological detection.

Some flexible biosensors have been recently fabricated from electrical and optical sensors^14–16^. Powerful LSPR biosensors can be fabricated as integrated platforms based on flexible substrates such as polydimethylsiloxane (PDMS)^17^, which has great acid–alkali resistance and high transparency. In metal–insulator–metal (MIM) structures, LSPR waves exhibit perfect optical absorption^18,19^ and are independent of the incident angle^20,21^ and polarization of light in the infrared (IR) region^22,23^. When light is incident on an MIM nanostructure, the resulting LSPR mode remains strongly confined in the structure^24^. In other words, the perfect absorber as sensor allows a wide angular range of incidence to utilize LSPR refractive index sensor operating under nonplanar surface. This concept provides a promising way forward to maintain the performance of LSPR sensors that can be easily carried and intimately paste with human body surfaces. The advantage of LSPR in MIM structures is that it makes them suitable for fabricating flexible biosensors. In general, the structure of MIM LSPR sensors are nonstretchable on a hard substrate^25,26^ and the sensitivity of LSPR refractive index sensors is below 500 nm/RIU^27–29^. Because the spatial distribution of the resonant mode in the geometric structure of traditional MIM LSPR sensor weakly overlaps with environmental refractive index to reduce the sensitivity of this sensor. The sensing performance of the MIM LSPR sensor can be improved by changes in its geometric shape and structure to enhance the sensitivity of flexible MIM LSPR sensor.

**Figure 1:**
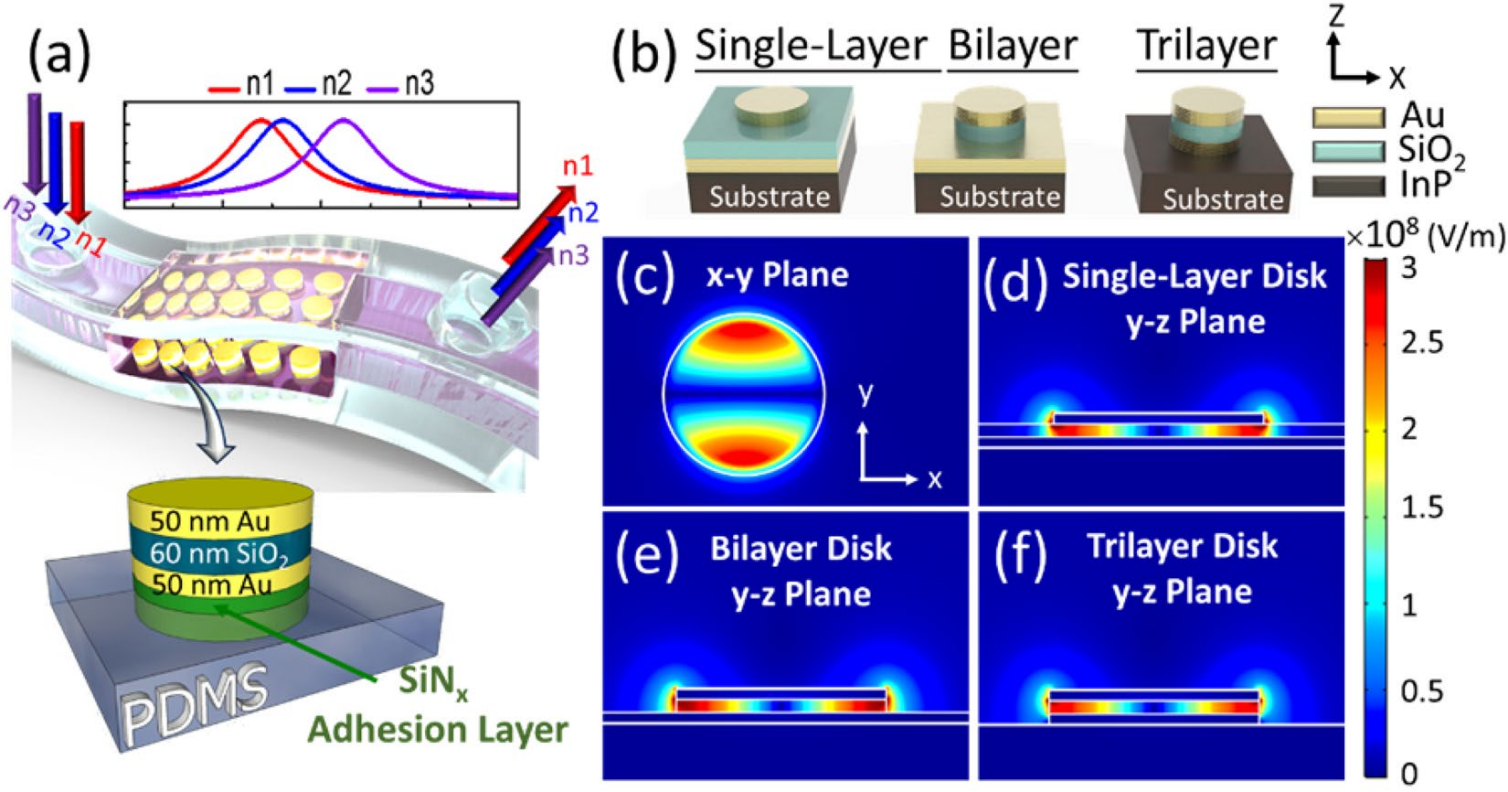
(**a**) Schematic diagram of the flexible MIM-disk LSPR refractive index sensor integrated in a PDMS fluidic chamber. A single MIM disk on a PDMS substrate is also shown. The MIM structure contains a 50-nm-thick Au disk, a 60-nm-thick SiO_2_ disk, a 50-nm-thick Au disk, and a 240-nm-thick SiN_x_ adhesion layer. (**b**) Structure diagrams of single-, bilayer-, and trilayer-MIM-disk LSPR sensors. (**c**) The mode profile of the electric field |E|^2^ of the MIM-disk LSPR sensor in the x-y plane. The mode profile of the electric field |E|^2^ of the (**d**) single-layer-, (**e**) bilayer-, and (**f**) trilayer-MIM-disk LSPR sensors in the y-z plane.

In this study, we demonstrated a flexible LSPR sensor containing a trilayer MIM disk reliably embedded in a PDMS substrate. High sensitivity for this LSPR sensor was achieved by varying the embedment depth of the trilayer MIM disk. In addition, wide-angle absorption in the MIM disk was beneficial for maintaining the device sensitivity stable under various bending curvatures. The schematic diagram and design of the flexible trilayer-MIM-disk LSPR sensor is shown in Fig. 1(a). In the future, flexible on-chip microfluidic biosensors can be developed by integrating LSPR sensors on chips capable of having multiple parallel channels on nonflat surfaces.

## Results

A periodic array of nanostructured trilayer MIM disks was designed for embedding into a flexible PDMS substrate by using a SiN_x_ adhesion layer. Figure 1(a) shows the schematic diagram and design of the stretchable trilayer-MIM-disk LSPR biosensor on a flexible PDMS substrate; the structure of a single MIM disk is also shown. Furthermore, this sensor can be integrated in a PDMS fluidic chamber for obtaining an on-chip microfluidic biosensor operating on a nonplanar surface. The MIM LSPR sensor is highly sensitive to changes in the environmental refractive index. When a fluid containing biological objects is injected into the sensor, a clear resonance peak shift is caused on varying the environmental refractive index. Moreover, the sensing performance of the MIM LSPR sensor is less influenced by variation in its geometric shape and structure^30–32^. Therefore, the MIM LSPR sensor can be used to detect changes in the environmental refractive index if the relationship between the geometric structure of the sensor and the spatial overlap of the resonant mode is known^33,34^. For determining this relationship, the resonant mode profiles for the three MIM disk structures were obtained using a finite element method numerical simulation. The disk diameter used in the simulation was 1600 nm. The simulated peak wavelength of the single-layer, bilayer, and trilayer-MIM-disks LSPR sensor were approximately 5.32, 5.11, and 5.09 μm, respectively. The different MIM-disk LSPR sensor geometries attribute resonant wavelength shift to change in the effective index (n_eff_).

The device structure diagrams and mode profiles of the electric field |E|^2^ for single-layer-, bilayer-, and trilayer-MIM-disk LSPR sensors are shown in Fig. 1(b–f). All the three sensor structures showed LSPR mode characteristics in the form of a strong resonance at the center of the disks. This observation implies that the LSPR wave was well confined in the MIM structures. The incident light induced charge on the Au nano-disk surface. The charges act as like an induced dipole, forming the localized surface plasmon polariton (LSPP). The LSPP at the metal/dielectric/metal cavity led to a strong coupling between the two LSPP modes and small cavity, thereby yielding a linear combination of two exponentially decaying LSPPs at the two Au/SiO_2_ interfaces. This strong coupling formed Fabry–Pérot-like resonances in the SiO_2_ cavity^18,24,35^.

According to the obtained mode profiles, the |E|^2^ field of the single-layer MIM disk was mostly localized in the SiO_2_ layer. However, a minor resonance wave was found to leak from the MIM disk into the external environment. The |E|^2^ field outside the MIM disk structure influenced the sensitivity of detecting resonance wavelength shifts by causing changes in the environmental refractive index. Therefore, when the SiO_2_ disk was removed, the |E|^2^ field increased and leaked into the environment. On removing the bottom Au layer, the field remained confined in the SiO_2_ cavity and the highest ratio of field leakage was observed for the trilayer disk MIM structure, as shown in Fig. 1(f). The LSPR wave was well confined in the trilayer disk MIM structure; moreover, its |E|^2^ field overlapped with the environment, thereby providing greater sensitivity for detecting resonance wavelength shifts.

**Figure 2:**
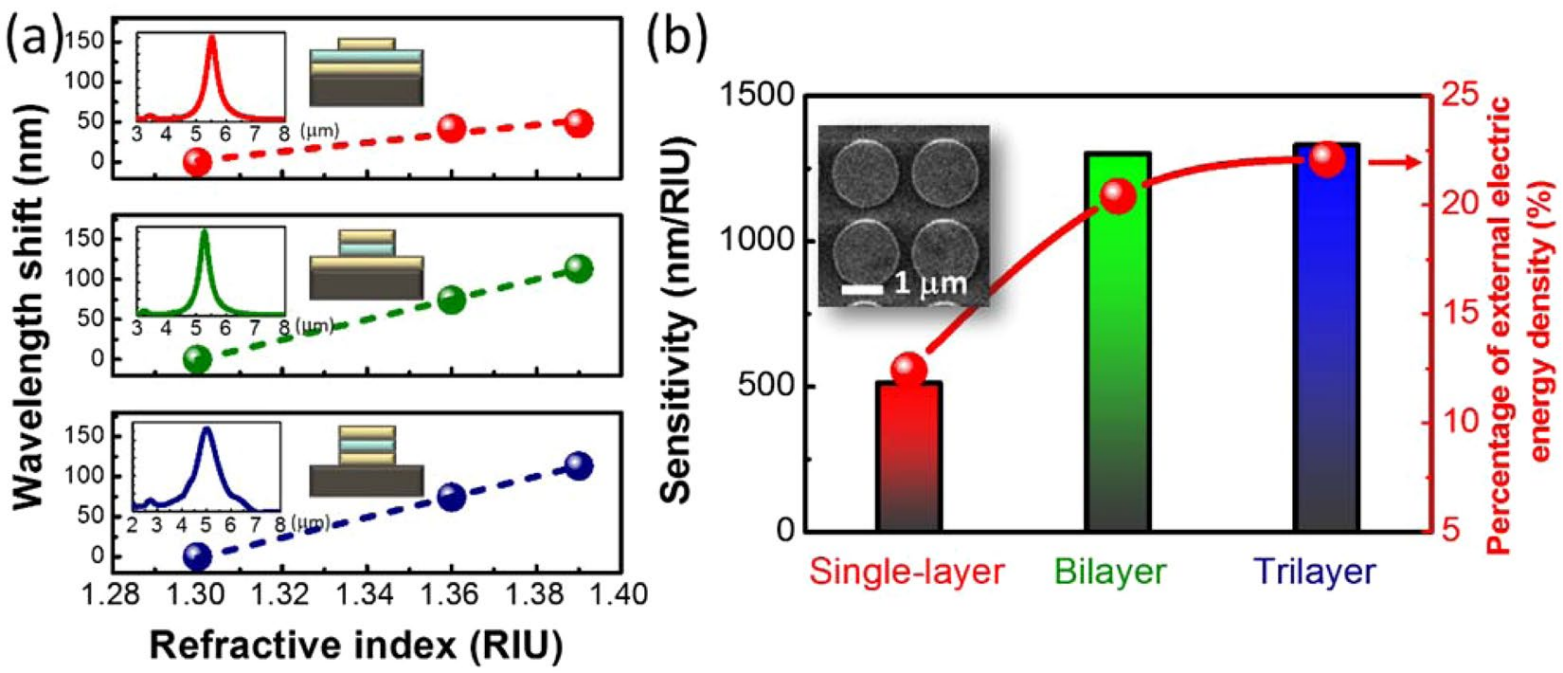
(**a**) The absorption spectrum and resonance wavelength shift for the three types of MIM-disk LSPR sensors as a function of refractive index. The wavelength shift of return-to-zero is obtained when the three types of sensors are covered with a liquid of refractive index n = 1.3. (**b**) Sensitivity and percentage of external electric energy density for the three types of MIM-disk LSPR sensors. Inset figure is top-view scanning electron microscopy image of the trilayer-MIM-disk LSPR sensor. The diameter of the disks is approximately 1600 nm.

Three types of Au/SiO_2_/Au MIM disks were originally fabricated on indium phosphide (InP) substrates. The different MIM disk structures were modified by controlling the ion-milling etching time. Subsequently, three types of MIM-disk LSPR sensors were fabricated. Inset figure in Fig. 2(b) shows the top view of a scanning electron microscopy image of the trilayer MIM disk array. The period was approximately 2000 nm, and the disk diameter was 1600 nm. The sensitivities of the three types of structures were characterized by obtaining their absorption spectra, for which different liquids with indices n = 1.30, 1.36, and 1.39 were dripped on top of the disk arrays. The peak wavelength of the single-layer, bilayer, and trilayer-MIM-disk LSPR sensor were approximately 5.5, 5.3 and 5.0 μm, respectively as shown inset figure in Fig. 2 (a). The LSPR peak shifted toward short wavelengths on increasing the etching depth. When the SiO_2_ layer and the bottom Au layer from the MIM disk structure were removed, the n_eff_ of the MIM structure was reduced and a blueshift was observed in the major LSPR resonance.

Figure 2(a) shows the LSPR peak shift as a function of environmental refractive index for the three types of sensors. The peak wavelength of the single-layer MIM disk array shifted by approximately 48 nm when the environmental refractive index changed from 1.30 to 1.39. The wavelength shift (Δλ), defined as the change in wavelength with respect to the wavelength measured for the environmental refractive index (n = 1.3), was calculated for different indices. Sensitivity is defined as the ratio of Δλ to Δn—change in the refractive index. For the three different etching depths of the three MIM disk structures, the sensitivity increased from 510 nm/RIU (refractive index unit) to 1330 nm/RIU, as shown in Fig. 2(b), with the trilayer MIM disk structure showing the highest sensitivity.

In order to determine the effect of |E|^2^ field overlapped with the environmental index refraction, the ratio of external electric energy density ${({\rm{W}}}_{{\rm{e}}}=\frac{1}{2}{{\epsilon }}_{i}{{\epsilon }}_{0}{E}^{2})$ is calculated using $\frac{{W}_{out}}{{W}_{total}}=\frac{\int \int {\int }_{V}^{V^{\prime} }{W}_{e}dV}{\int \int {\int }_{V}^{V}{W}_{e}dV+\int \int {\int }_{V}^{V^{\prime} }{W}_{e}dV}$. Here, *V* is the volume of the MIM disk device and *V*′ is the volume of the outside electric field. ${{\epsilon }}_{i}$ and ${{\epsilon }}_{0}$ are relative permittivity and vacuum permittivity, respectively. The percentage of external electric energy density for single-layer, bilayer, and trilayer MIM disk structures was estimated to be approximately 12.4, 20.4, and 22.1%, respectively. Percentage of external electric energy density for the three types of MIM-disk LSPR sensor also presents in Fig. 2 (b). The percentage of external electric energy density for bilayer and trilayer MIM disk structures were both higher than that for the single-layer MIM disk structure. In addition, the trilayer MIM disk structure had the highest field overlapping. Because the spatial overlap of the resonant mode with the environment increased for the different geometric structures of the MIM LSPR sensors. A positive correlation can be observed between the sensitivity enhancement and the percentage of external electric energy density. The structural characteristics of the sensors were optimized on the basis of the highest sensitivity toward changes in the environmental refractive index by simulating the absorption spectra and resonant mode profiles of the sensors.

**Figure 3:**
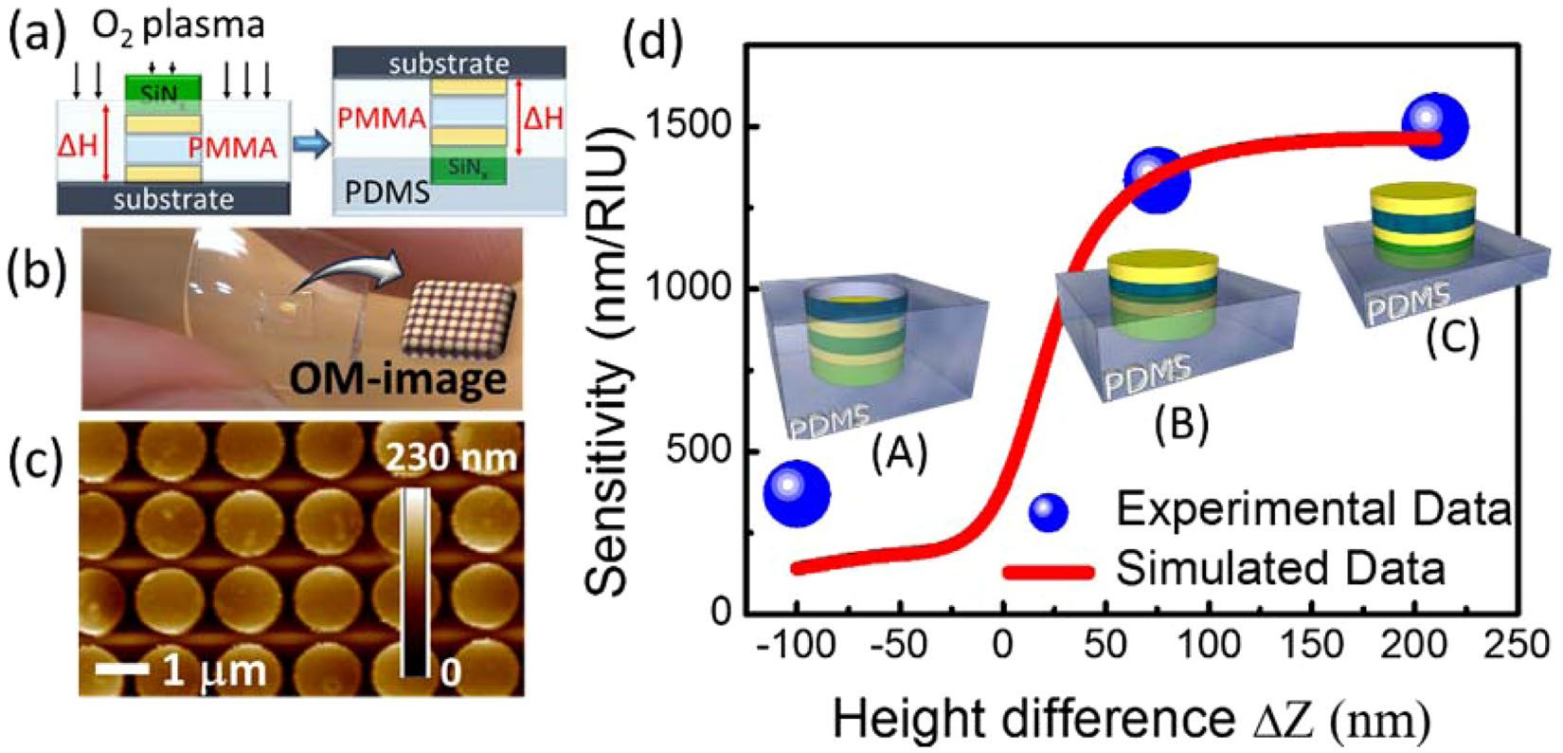
(**a**) Schematic of the substrate transfer process and the method for controlling the bonding depth of the MIM disk on the PDMS substrate. The height (∆H) of the MIM disk exposed to the environment was controlled by O_2_ plasma etching the thickness of the PMMA. Subsequently, the samples were bonded into PDMS. (**b**) The flexible MIM-disk LSPR sensor on a PDMS substrate. The inset figure is an optical microscopy image of the MIM-disk LSPR sensor. (**c**) The AFM surface topography for sample C with a PMMA etching time of 5 min. (**d**) Sensitivity of the MIM-disk LSPR sensors on a flexible PDMS substrate with different bonding depths. The height difference is defined as the difference between the heights of the PDMS surface and the top surface of the MIM disk. The top surface of the MIM disk is used as a reference. The height difference for samples A, B, and C of the MIM-disk LSPR sensor with disks embedded in the PDMS is −100 nm, 75 nm, and 200 nm, respectively.

As the next step, the trilayer MIM disk array of the LSPR sensors was transferred to a flexible PDMS substrate by directly bonding the array into PDMS through a 240-nm-thick SiN_x_ adhesion layer. To increase the sensitivity of the LSPR sensor and avoid the absorption of PDMS substrate, it is important to vary the bonding depth of the MIM disk by controlling the height difference between the PDMS surface and the MIM section of the LSPR sensor. Because the bonding depth of the MIM disk on the flexible substrate varied, the MIM section of the sensor exposed to the environment was controlled by controlling the thickness of PMMA. This step also prevents the optical absorption of PDMS substrate and enhances the visibility of LSPR sensing signals. The schematic diagram of the substrate transfer process and the method for controlling the bonding depth of the MIM disk on the PDMS substrate are shown in Fig. 3(a). The flexible MIM-disk LSPR sensor on the PDMS substrate was fabricated as shown in Fig. 3(b). The height difference (ΔZ) between the top surfaces of PDMS and the MIM disk was calculated through atomic force microscopy (AFM). The scanned surface area was approximately 15 μm × 15 μm. The average ΔZ value for sample A with a PMMA etching time of 7 min was −100 nm. This implies that the MIM disk was fully embedded in the PDMS substrate. Because the thickness of the PMMA layer determined the starting point of PDMS coverage on the MIM disk structure, ΔZ between the PDMS surface and top of the sensor increased with decreasing PMMA etching time. ΔZ needed to be optimized for correctly exposing the MIM disk to the environment to increase sensitivity. The ΔZ for samples B and C was 75 and 200 nm, respectively, with PMMA etching times of 6 and 5 min, respectively. The ΔZ value between the top surface of PDMS and the MIM disk for sample C was approximately 200 nm as shown in Fig. 3(c). This result indicates that for sample B, the top of the Au layer and half of the SiO_2_ layer were exposed to the environment, whereas for sample C, the whole MIM structure was exposed. The performance of the MIM LSPR sensors with different bonding depths is shown in Fig. 3(d). The experimental sensitivity of MIM disks on a PDMS substrate as a function of the exposed height was calculated and compared with the simulation results. The sensitivity of samples A, B, and C was approximately 210, 1330, and 1500 nm/RIU, respectively. Thus, the increase in sensitivity strongly depended on the exposed height of the MIM disk structure. The sensitivity of the MIM disk on the flexible PDMS substrate increased with an increase in the exposed height. Thus, the sensitivity of sample C was 7.5 times that of sample A. The simulation results demonstrated that the sensitivity rapidly increased when the exposed height was in the range of 0–150 nm. Because the combined sensitivity increased because of MIM disk exposure and environmental contribution led to greater detection, the resonant wavelength shifted with changes in the environmental refractive index.

**Figure 4:**
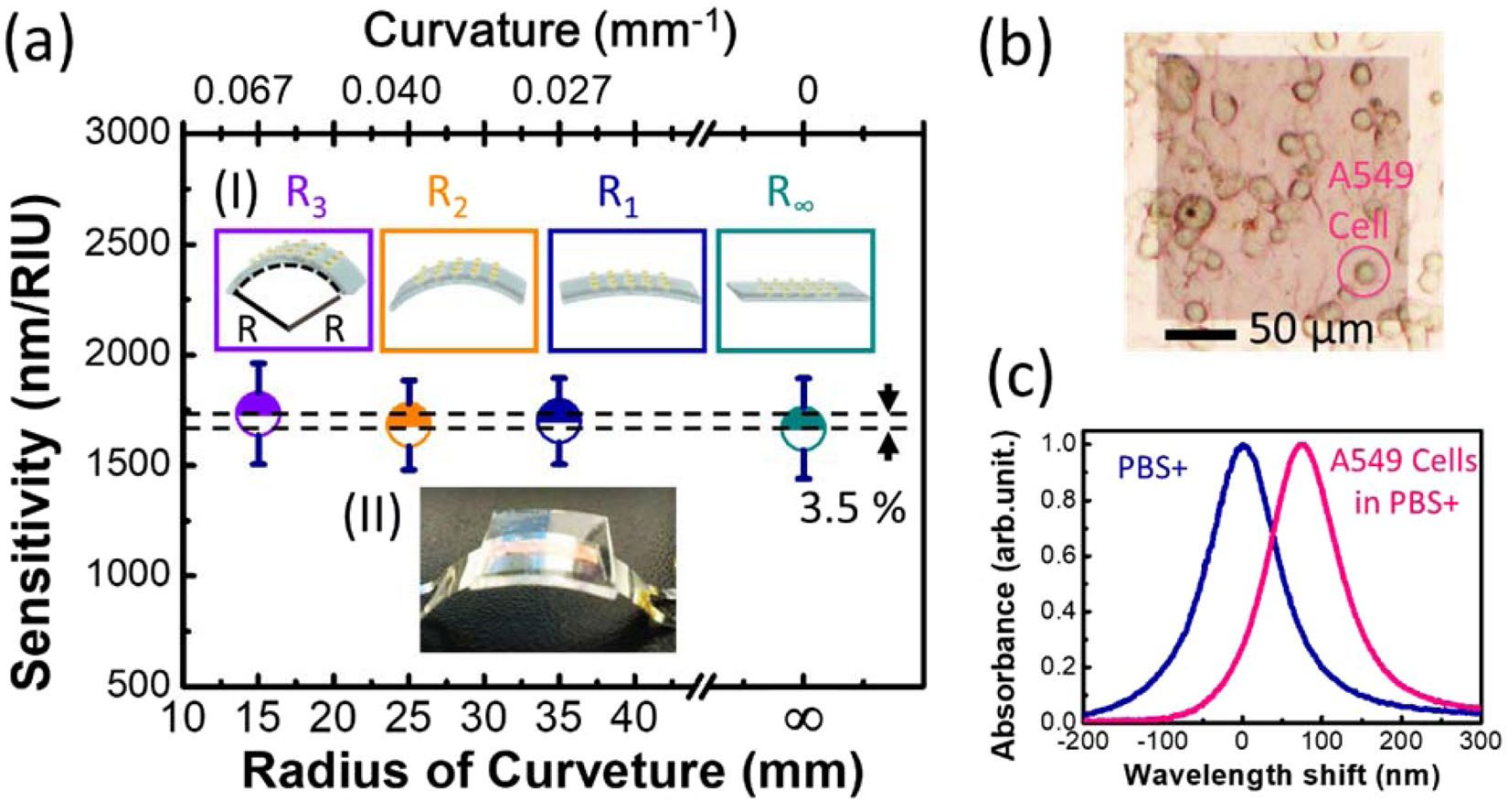
(**a**) Sensitivity of MIM-disk LSPR sensor on a flexible substrate with the curvature of the surface varying from 1/R_∞_ = 0 to 1/R_3_ = 0.066 mm^−1^ (R_1_ = 35 mm, R_2_ = 25 mm, and R_3_ = 15 mm); here, R is the radius of curvature. Inset figure (I) is the schematic diagram of the sensor bending upon varying the radius of curvature. Inset figure (II) shows the bending of the MIM-disk LSPR sensor on a flexible substrate, to be fixed on a bending metal plate. (**b**) An optical microscopy image of living A549 human lung cancer cells in a phosphate buffered saline buffer solution as detected by the MIM-disk LSPR biosensor. (**c**) Absorption spectra of a phosphate buffered saline solutions with A549 cancer cells and phosphate buffered saline solution without cells, as detected using MIM-disk LSPR biosensors. The wavelength shift of return-to-zero is based on the absorption spectra of the MIM-disk LSPR biosensors covered with a phosphate buffered saline solution.

After sensitivity analysis, the sensitivity stability of the flexible MIM-disk LSPR sensor on a nonplanar substrate was also analyzed, as shown in Fig. 4(a). In this sensor on a nonplanar substrate, an angle of inclination and a slight deformation of the substrate could be induced. For creating flexible sensors, understanding the influence of the bending-inducing mechanism is important. The flexible MIM-disk LSPR sensor was placed on surfaces with different curvatures, and its sensitivity was measured for analyzing its sensitivity stability. Inset (I) in Fig. 4(a) shows the schematic diagram of a bending sensor with its curvature varying from 1/R_∞_ = 0 to 1/R_3_ = 0.066 mm^−1^ (R_1_ = 35 mm, R_2_ = 25 mm, and R_3_ = 15 mm), where R is the radius of curvature. A series of bent metallic plates with varying curvatures were fabricated, and the sensor was fixed on these plates by using a double-sided tape for sensitivity measurement, as shown in Fig. 4(a), inset (II). Figure 4 shows the sensitivity of the MIM-disk LSPR sensor on a PDMS substrate with gradually increasing curvature. The sensitivity of the optimized sensor on a planar surface was approximately 1670 nm/RIU in air (n = 1). The experimental sensitivity of this sensor varied between 1670 and 1730 nm/RIU with curvature varying from 1/R_∞_ to 1/R_3_. The sensitivity remained stable as the sensitivity variation was less than 3.5% (±1.75%) under different bending curvatures. Thus, flexible MIM LSPR sensors on nonplanar substrates have stable sensing performance. The advantage of fabricating an LSPR sensor on an MIM structure is that this structure exhibits perfect optical absorption and permits a wide range of incident angles in the IR region^36^. Because the LSPR resonant mode is strongly confined in the MIM structure, the LSPR wave characteristics make the MIM structures suitable candidates for use in flexible biosensors. The slight variation in sensitivity of the MIM LSPR sensor with varying bending curvature of the MIM disk could be caused by experimental measurement error or the different bonding depths of MIM disks on the PDMS substrate in a cell array. Moreover, the embedment depth of MIM disks on the PDMS substrate can vary with the distribution of surface inclination, thereby slightly increasing or decreasing sensitivity. The sensitivity error was less than 3.5% under different bending curvatures. Because this small error would not cause problems during detection, it can be ignored. Therefore, even on a bending substrate, the flexible MIM-disk LSPR sensor has high sensitivity as well as high sensitivity stability under different bending curvatures. The flexible MIM-disk LSPR sensor on nonplanar substrates can be used to fabricate flexible biosensors for disease detection. It is worth to note that the sensitivity of the flexible LSPR MIM-disk array was investigated under the convex bending in the study. The optical coupling between the LSPR waves might take some effects under the concave bending conditions if the periodicity is too small. The LSPR resonances in the MIM nanodisk are mainly formed due to the strongly confinement between the Au/SiO_2_/Au interfaces and is independent to the periodicity^37^. The coupling between the adjacent disks might take effects if the period of MIM-disk array smaller than 2 μm.

In the current study, the MIM-disk LSPR sensor was used for detecting A549 cancer cells in a phosphate buffered saline (PBS+) solution^38,39^. A549 human lung cancer cells are critical for cancer study and are also used to test the sensing abilities of LSPR sensors for cell detection. Determine changes in the refractive index for A549 cells in a buffer solution, such as PBS+, is difficult because the refractive index of the cells differs only a little from that of buffer solutions^40^. Figure 4(b) shows an optical microscopy image of living A549 cells with radioactive-labeled cell surface on the MIM disk array of the LSPR biosensor. The array size was 200 μm × 200 μm. The sensitivity of the sensor was 1670 nm/RIU. To avoid the absorption due to the PBS+ solution, in this work, we choose the working wavelength of the LSPR MIM sensors in the regimes of 5–6 μm. Figure 4(c) illustrates the absorption spectra of MIM-disk LSPR biosensors covered in the PBS+ solution containing A549 cancer cell seeding (pink line) and PBS+ solution without any cell seeding (blue line) with label-free detection. The wavelength shift of return-to-zero was based on the absorption spectra of MIM-disk LSPR biosensors covered in the PBS+ solution. For A549 cancer cells in the PBS+ solution, the absorption spectra of the MIM-disk LSPR biosensor shifted by 75 nm. Therefore, this biosensor was highly sensitive and could easily distinguish the refractive index change between A549 cancer cells and the PBS+ solution for the detection of cancer cells. Because the A549 human lung cancer cells were added to the environment (PBS+ buffer solution), a small local refractive index change occurred. The sensitivity variation (±1.75%) of the flexible MIM-disk sensor might be visible with the peak wavelength shift (75 nm) during A549 cell detection. This could be much improved by optimizing the flexible MIM LSPR sensor structure and device fabrication in the future. The LSPR biosensor had a high intensity and strongly localized electromagnetic field, which could act as an extremely sensitive probe to detect even small changes in the locally surrounding dielectric medium. This verified cell attachment to the disks because the field was locally confined for LSPR, thus demonstrating the cell detection ability of the trilayer-MIM-disk LSPR sensor. This highly sensitive MIM-disk LSPR biosensor is a powerful device for *in vitro* monitoring of cancer cells during drug testing. During the sensing process, incident light in the IR region is used for detecting optical changes. This light does not harm living cells; thus, the MIM disk array is an ideal candidate for use in biosensors. Therefore, the MIM-disk LSPR sensor was used for the real-time detection of A549 cancer cells in the PBS+ solution. This LSPR sensor is advantageous in the mid-IR wavelength region because of its ability to sense optically without causing damage to living cells. At current stage, we focus on the design and optimization of a flexible MIM LSPR sensing structure, and demonstrate the cell sensing capability. The detection of cells with different coverage ratios would be one of important applications for future biomedical applications.

## Conclusion

In summary, we demonstrated a LSPR sensor with MIM disks embedded into a flexible PDMS substrate through a SiN_x_ adhesion layer. The sensitivity of this sensor was approximately 1670 nm/RIU, which remained stable (sensitivity variation was less than 3.5%) under different bending curvatures_._ The trilayer MIM disk structure of the LSPR sensor showed high sensitivity owing to the spatial overlap of the LSPR wave with the environment. The LSPR resonant mode was strongly confined in the MIM structure; the LSPR wave characteristics make this structure a suitable candidate for fabricating flexible biosensors. Furthermore, the MIM-disk LSPR sensor was used for detecting A549 cancer cells in a PBS+ solution. The absorption spectra of the MIM-disk LSPR sensor clearly shifted by 75 nm, easily distinguishing between PBS+ solutions with A549 cancer cells, thus aiding label-free monitoring of cancer cells. The flexible MIM-disk LSPR sensor had high sensitivity as well as high sensitivity stability under different bending curvatures. Moreover, this sensor can be integrated in a PDMS fluidic chamber for fabricating an on-chip microfluidic biosensor. The flexible MIM-disk LSPR sensor on nonplanar substrates can be used to fabricate flexible biosensors for detecting several major diseases, such as cancer.

## Methods

### Flexible MIM-disk LSPR sensor preparation

The fabrication process of the MIM-disk LSPR sensor is described here. For fabricating the MIM structure, a 50-nm-thick Au film, a 60-nm-thick SiO_2_ layer, and a 50-nm-thick Au film were deposited through electron-beam physical vapor deposition (by using an e-gun system) and plasma-enhanced chemical vapor deposition. A 240-nm-thick SiN_x_ layer, deposited on top of the MIM structure, acted as a hard mask for dry etching. Patterns were defined and transferred to the SiN_x_ layer through electron beam lithography and inductively coupled plasma/reactive ion etching, respectively. The MIM disk structures were formed using ion-milling etching. Before substrate transfer, the MIM disk was covered with a 950 PMMA A5 photoresist layer through spin coating. The PMMA layer was then etched to control its thickness by controlling the etching time. The etching rate of 950 PMMA A5, etched using oxygen plasma at 30 W, was approximately 0.83 nm/s. A flexible PDMS substrate was prepared by mixing Sylgard 184 A and B in a 10:1 volume ratio. The PDMS substrate was baked at 60 °C for 15 min. The MIM disk on InP with a PMMA coating was bonded onto the PDMS substrate with the upside. Finally, the sample was immersed in a dilute HCl solution at room temperature for about 60 min to remove InP. After InP removal, PMMA was cleaned by immersing the sample in an acetone solution.

### Cell growth and fixation

The MIM disk device was immersed in PBS+ solution with A549 cells. In the cell experiments, cell suspension with density of 5 × 10^5^ cell/ml was prepared and dispensed on top of the sensing area. The A549 cells adhered onto the device surface within 3 hours under the static culture condition. After cell adhesion, we used PBS+ solution to wash out unattached cells to form a single layer of A549 cells on the device surface. The adhesion of the A549 cells was confirmed by microscopic imaging in the experiments.

### Characterization

The absorption spectra of the three types of MIM LSPR sensors were obtained on a Fourier-transform IR (FTIR) spectrometer (Bruker VERTEX 70). Mid-IR (near-IR) light was focused on the samples and was reflected back through a 15x objective lens. The back and reflected light were passed through a 200 μm × 200 μm slit, the same size as that of the disk array, and were detected on a mercury–cadmium–telluride photodetector with a spectral resolution of 2 cm^−1^. The absorption spectra of the MIM LSPR sensors on a hard substrate were obtained at room temperature.
